# Neuroanatomy of Individual Differences in Language in Adult Males with Autism

**DOI:** 10.1093/cercor/bhu211

**Published:** 2014-09-23

**Authors:** Meng-Chuan Lai, Michael V. Lombardo, Christine Ecker, Bhismadev Chakrabarti, John Suckling, Edward T. Bullmore, Francesca Happé, MRC AIMS Consortium, Declan G. M. Murphy, Simon Baron-Cohen

**Affiliations:** 1 Autism Research Centre, Department of Psychiatry, University of Cambridge, Cambridge CB2 8AH, UK; 2 Department of Psychiatry, National Taiwan University Hospital and College of Medicine, Taipei 10051, Taiwan; 3 Department of Psychology and Center for Applied Neuroscience, University of Cyprus, Nicosia CY 1678, Cyprus; 4 Sackler Institute for Translational Neurodevelopment, Department of Forensic and Neurodevelopmental Sciences, Institute of Psychiatry, King's College London, PO23, Institute of Psychiatry, London SE5 8AF, UK; 5 School of Psychology and Clinical Language Sciences, Centre for Integrative Neuroscience and Neurodynamics, University of Reading, Reading RG6 6AL, UK; 6 Brain Mapping Unit, Department of Psychiatry, University of Cambridge, Cambridge CB2 0SZ, UK; 7 GlaxoSmithKline, Clinical Unit Cambridge, Addenbrooke's Hospital, Cambridge CB2 2QQ, UK; 8 Cambridgeshire and Peterborough NHS Foundation Trust, Cambridge CB21 5EF, UK; 9 MRC Social, Genetic and Developmental Psychiatry Centre, Institute of Psychiatry, King's College London, PO80, Institute of Psychiatry, London SE5 8AF, UK

**Keywords:** autism, individual differences, language, neuroanatomy, specifiers

## Abstract

One potential source of heterogeneity within autism spectrum conditions (ASC) is language development and ability. In 80 high-functioning male adults with ASC, we tested if variations in developmental and current structural language are associated with current neuroanatomy. Groups with and without language delay differed behaviorally in early social reciprocity, current language, but not current autistic features. Language delay was associated with larger total gray matter (GM) volume, smaller relative volume at bilateral insula, ventral basal ganglia, and right superior, middle, and polar temporal structures, and larger relative volume at pons and medulla oblongata in adulthood. Despite this heterogeneity, those with and without language delay showed significant commonality in morphometric features when contrasted with matched neurotypical individuals (*n* = 57). In ASC, better current language was associated with increased GM volume in bilateral temporal pole, superior temporal regions, dorsolateral fronto-parietal and cerebellar structures, and increased white matter volume in distributed frontal and insular regions. Furthermore, current language–neuroanatomy correlation patterns were similar across subgroups with or without language delay. High-functioning adult males with ASC show neuroanatomical variations associated with both developmental and current language characteristics. This underscores the importance of including both developmental and current language as specifiers for ASC, to help clarify heterogeneity.

## Introduction

The 5th edition of the Diagnostic and Statistical Manual of Mental Disorders (DSM-5) collapsed the DSM-IV subtypes of autism (autistic disorder, Asperger's disorder, childhood disintegrative disorder, and pervasive developmental disorder not otherwise specified) into a single diagnosis called “autism spectrum disorder” (ASD) (American Psychiatric Association 2013). These changes were based on the claim that DSM-IV subtypes are not reliably differentiated by clinicians (Lord et al. 2012). DSM-5 argued that a unitary label eliminates diagnostic confusion surrounding subtypes, especially between Asperger's disorder/syndrome (AS) and high-functioning autistic disorder (HFA) (Happé 2011).

However, the elimination of diagnostic subtypes (lumping) does not resolve the problem of high heterogeneity (Lai, Lombardo, Chakrabarti et al. 2013; Waterhouse 2013). In fact, the reverse may be true: fractionating phenotypes into subgroups is still needed to understand the biological basis of “the autisms,” and to identify valid and reliable biomarkers (Lord and Jones 2012; Murphy and Spooren 2012; Ecker, Spooren et al. 2013; Grzadzinski et al. 2013; Lai, Lombardo, Chakrabarti et al. 2013; Tsai and Ghaziuddin 2014). There is thus a need to move forward from investigating average differences between individuals with and without an ASD diagnosis, to also identify key dimensions of individual differences “within” the spectrum.

DSM-5 now includes “specifiers” for ASD, which is a useful starting point to delineate individual differences in autism spectrum conditions (ASC: hereafter we use this term as a preferred synonym for ASD, to avoid the pejorative implications of the word “disorder”). Though no longer listed as a required symptom, anomalies in the structural properties of language (including the nonpragmatic aspects such as phonology, syntax, morphology, and semantics; for brevity, this is simply referred to below as “language”) have long been considered central to ASC, even in individuals with an average or above-average IQ (Boucher 2012; Lord and Jones 2012). One of the specifiers suggested by DSM-5 is “(currently) with or without accompanying language impairment” (American Psychiatric Association 2013). We have argued elsewhere (Lai, Lombardo, Chakrabarti et al. 2013) that “both” historical/developmental and current language are likely to be key factors contributing to individual differences, and should both be investigated at multiple levels, from cognition to neurobiology.

One longstanding issue in autism research is “does delayed language development matter?” DSM-5 eliminated developmental cutoffs that previously distinguished AS from HFA, but debate still exists over whether AS (who by definition have no language delay) and HFA (who by definition have language delay) are distinct diagnostic categories (Gillberg 1998; Kugler 1998; Frith 2004). Studies have not provided a conclusive answer at either clinical, behavioral or cognitive levels (Tsai 2013), and suggest that the observed group differences are mainly associated with variations in age, intellectual ability, and expressive language (Macintosh and Dissanayake 2004; Witwer and Lecavalier 2008). Furthermore, studies investigating the discriminant validity of AS versus HFA often suffer from inconsistent diagnostic definition: Some give precedence to HFA over AS (as suggested by DSM-IV), some give precedence to AS over HFA and focus more on whether language/cognitive delay is present, and some rely on additional diagnostic features not included in DSM-IV criteria (Klin et al. 2005; Witwer and Lecavalier 2008). Studies also often suffer from circularity because the dependent measures are used to derive the diagnostic assignment (Witwer and Lecavalier 2008). It is therefore important to move away from asking whether AS and HFA are distinct categories, to simply ask: “Do individuals on the autism spectrum vary (on some independent measures) as a function of their language developmental history?”

Although there are a number of behavioral and cognitive studies, with inconsistent results, only a few small-scale studies have investigated the neuroanatomical correlates of early language development in autism, mostly in children and adolescents (Kwon et al. 2004; Lotspeich et al. 2004; McAlonan et al. 2008, 2009; Toal et al. 2010); see Supplementary Text for a summary. These studies aimed to differentiate AS from HFA/autistic disorder, but the 2 groups were actually defined purely by the absence or presence of language delay (based on first words/phrases), using specific developmental cutoffs. They all demonstrate group differences, but the patterns are inconsistent. Two meta-analyses additionally provide relevant but only indirect information. One contrasted summary findings from voxel-based morphometry (VBM) studies (ASC vs. controls) with the majority of ASC individuals (>70%) having a history of language delay with summary findings from studies where the majority of ASC individuals had no language delay, and noted that areas identified by the 2 study sets were largely distinct in terms of location and the directionality of differences (Yu et al. 2011). However, a direct meta-analytic comparison between groups of ASC individuals with versus without language delay is not yet possible because there have been too few studies providing relevant data. Another meta-analysis found no statistically significant effects of an AS diagnosis versus other ASC diagnoses and concluded that AS and autistic disorder share similar neural substrates (Via et al. 2011). The analysis, however, was limited to brain regions showing significant differences between all individuals with ASC versus controls, which may have missed significant differences outside of these confined regions.

Regarding current language, although the neuroanatomical basis of autism has been associated with systems underlying the structural properties of language (Groen et al. 2008; Wan and Schlaug 2010), most studies that test this hypothesis have only compared groups with or without autism and at the functional level, showing atypical neural activation and synchronization (Harris et al. 2006; Kleinhans et al. 2008; Catarino et al. 2011; Tesink et al. 2011; Beacher et al. 2012; Eyler et al. 2012; Lai, Pantazatos et al. 2012; Kenworthy et al. 2013; Lo et al. 2013; Williams et al. 2013). A few studies have examined the neuroanatomical correlates of current language ability in individuals with ASC using diffusion imaging (Fletcher et al. 2010; Lange et al. 2010; Nagae et al. 2012; Verhoeven et al. 2012; Lo et al. 2013; Verly et al. 2014), and some show associations between impaired white matter (WM) microstructural properties of language pathways and poorer language ability. Some other studies have investigated the relationship between language and volume (or asymmetry) in specific cortical gray matter (GM) language-processing regions (De Fosse et al. 2004; Bigler et al. 2007) and the cerebellum (Hodge et al. 2010). Overall these studies have only examined confined brain regions. For a more comprehensive investigation, in the current study, we used a hypothesis-free, whole-brain approach to study individual differences in ASC in neuroanatomy as a function of current language ability.

The present study investigated whether one source of heterogeneity within the autism spectrum is individual differences in language and associated neuroanatomy. Within a large cohort of male adults with ASC, we tested how individuals vary as a function of language developmental history and current structural language measures. Our question was not how individuals with ASC differ from the “neurotypical” population in average. For this reason, a neurotypical group was not included in the main analyses on within-ASC variability. Neuroanatomical comparisons of ASC versus neurotypical control groups have been reported elsewhere (Ecker et al. 2012; Ecker, Ginestet et al. 2013). However, clarifying the commonality shared by subgroups is also important for our conceptualization of the ASC category. A subsidiary analysis was thus performed to include an additional age- and IQ-matched neurotypical group to test the commonality in volumetric features (measured by spatial overlap) between 1) the group-difference map of ASC individuals with language delay versus neurotypical individuals and 2) the group-difference map of ASC individuals without language delay versus neurotypical individuals (i.e., between the so-called HFA-neurotypical and AS-neurotypical differences). This informs the commonality within ASC, despite variations in language development, in parallel to the investigation of heterogeneity.

We first investigated the neuroanatomical correlates of a history of language delay using VBM, where “delay” was defined in a binary fashion, in line with the majority of previous neuroimaging (Kwon et al. 2004; Lotspeich et al. 2004; McAlonan et al. 2008, 2009; Toal et al. 2010) and behavioral studies (Howlin 2003; Klin et al. 2005). This was followed by subsidiary spatial overlap analyses (using an additional neurotypical group for contrast) testing for commonality between those with and without delay. Second, we identified the neuroanatomical correlates of a latent variable (LV) of current language ability, using Partial Least Squares (PLS) analysis. PLS is a multivariate technique that employs singular value decomposition on a correlation matrix of brain and behavioral variables, in order to extract latent brain and behavioral variable pairs with high degrees of covariance (Krishnan et al. 2011). We chose PLS because of the distributed nature of neural systems involved in language (Friederici 2012), and because we were interested in the common latent factor underlying available language measures, rather than specific aspects of language.

## Materials and Methods

### Participants

Eighty right-handed Caucasian adult males with ASC (aged 18–41 years) participated as part of the UK Medical Research Council (MRC) Autism Imaging Multicentre Study (AIMS). Recruitment details are reported elsewhere (Ecker et al. 2012; Ecker, Ginestet et al. 2013). Data were collected from 3 centers: the Institute of Psychiatry, King's College London (KCL) (*n* = 36); the Autism Research Centre, University of Cambridge (*n* = 28); and the Autism Research Group, University of Oxford (*n* = 16). All participants had a formal clinical diagnosis of autistic disorder or Asperger's disorder (Asperger's syndrome) based on DSM-IV (American Psychiatric Association 2000) or ICD-10 (World Health Organization 1992) criteria, from a psychiatrist or clinical psychologist working in the UK National Health Service. Diagnosis was further confirmed using the Autism Diagnostic Interview-Revised (ADI-R) (Lord et al. 1994). To be included, participants had to score at or above the diagnostic algorithm cutoffs but were permitted to score 1 point below threshold in one of the 3 symptom domains. This allowed for possible underestimation of early developmentally atypical behavior in the recollection of caregivers whose children were now adults. Module 4 of the Autism Diagnostic Observation Schedule (ADOS) (Lord et al. 2000) was performed but the score was not used as an inclusion criterion due to low sensitivity for detecting high-functioning adults with autism (Lai et al. 2011). These procedures are the same as those used in our earlier studies (Lai et al. 2010, 2011; Lombardo et al. 2010, 2011; Ecker et al. 2012; Lai, Lombardo, Ruigrok et al. 2012; Ecker, Ginestet et al. 2013; Wilson et al. 2014).

Exclusion criteria included current or history of major psychiatric conditions (e.g., psychotic disorders, bipolar disorders, substance-use disorders), head injury, genetic syndromes, medical conditions affecting brain structure and function (e.g., epilepsy), intellectual disability (IQ < 70), Tourette's syndrome, hyperkinetic disorder, and use of antipsychotic medications or mood stabilizers. Depressive and anxiety disorders were not exclusion criteria due to their high prevalence (∼50%) in adults with autism (Hofvander et al. 2009; Cassidy et al. 2014). Eight individuals reported history of antidepressant use (4 with fluoxetine, 2 with paroxetine, 1 with sertraline, and 1 with amitriptyline). No individuals reported regular use of benzodiazepine or other anxiolytics.

For the subsidiary analysis (testing for commonality between so-called AS-neurotypical and HFA-neurotypical differences), we included data from an additional 57 male neurotypical participants from the control cohort of the MRC AIMS project (KCL *n* = 26, Cambridge *n* = 21, Oxford *n* = 10), that were matched in age and full-scale IQ with both ASC subgroups (with or without language delay). The exclusion criteria were identical, and additionally they did not have ASC themselves or in their family history. No neurotypical individuals reported use of antidepressant, benzodiazepine or other anxiolytics.

All participants gave informed written consent in accordance with the ethics approval from the National Research Ethics Committee, Suffolk, UK.

### Measures

#### Autism-Related Measures

The participant's main childhood caregiver was interviewed using the ADI-R (Lord et al. 1994). All participants were assessed using module 4 of the ADOS on the date of scanning (Lord et al. 2000). Participants also completed measures of self-reported autistic traits using the Autism Spectrum Quotient (AQ) (Baron-Cohen, Wheelwright, Skinner et al. 2001) and empathy using the Empathy Quotient (EQ) (Baron-Cohen and Wheelwright 2004). Advanced mentalizing ability was assessed with the “Reading the Mind in the Eyes” test (Eyes Test) (Baron-Cohen, Wheelwright, Hill et al. 2001).

#### Language Measures: Developmental History and Current Ability

“History of language development” was assessed as part of the ADI-R interview. The caregiver was asked about the age of participant's “first single words,” defined as “words used repeatedly and constantly for the purpose of communication with reference to a particular concept, object, or event,” excluding “mommy” and “daddy.” The caregiver was also asked about the age at which the participant started using phrases (age of first phrases), defined as 2 or more words including a verb. In accordance with standard clinical practice that categorically defines language delay in autism, as well as the common research definition (Howlin 2003; Kwon et al. 2004; Lotspeich et al. 2004; Klin et al. 2005; McAlonan et al. 2008, 2009; Toal et al. 2010), a positive history of language delay was defined either as having “first single words” later than 24 months, or an “age of first phrases” later than 33 months, or both.

“Current structural language ability” can be assessed across a wide range of measures. Given the purpose of obtaining a general estimation in this domain (i.e., reflecting basic cognitive processes central to structural language processing, rather than about specific language functions) and the limited testing time and loading that was acceptable for the ASC participants, we employed 3 general measures that are widely used, well validated, viable for individuals with ASC, and reflect basic aspects of cognitive processes in relation to structural language, rather than an exhaustive battery of specific measures (Wilson et al. 2014).

The verbal IQ (VIQ) from the Wechsler Abbreviated Scale of Intelligence (WASI) (Wechsler 1999) served as the first language measure. VIQ comprises 2 subtests: “Vocabulary,” which measures lexical knowledge and verbal concept formation, and “Similarities,” which measures verbal reasoning, semantic ability, and concept formation.

Second, word generativity was tested using the F-A-S task (Gladsjo et al. 1999). Participants are asked to produce as many words as possible within 1 min that begin with the letter “F”; the same instructions are repeated for words starting with the letters “A” and “S.” Names, tense changes, plurals, derivatives, and pronouns are not allowed. Total words generated, excluding repetitions and those breaking rules, are treated as the outcome measure.

Last, phonological memory was tested using the Non-Word Repetition (NWR) task (Gathercole et al. 1994). This consists of 28 nonwords (i.e., unfamiliar phonological items that conform to the phonotactic rules of English but do not exist in the English lexicon). Participants are asked to listen to a nonword and repeat it immediately. Their utterance is audio-recorded and coded as correct or incorrect on the basis that all vowels, consonants, and accents of the uttered repetition are exactly the same as the stimulus. Number of correct items is treated as the outcome measure.

### Image Acquisition and Preprocessing

Participants were scanned using 3 T MRI scanners fitted with an 8-channel receive-only RT head-coil: GE Medical Systems HDx, Department of Radiology, University of Cambridge, and Centre for Neuroimaging Sciences, Institute of Psychiatry, King's College London; Siemens Medical Systems Tim Trio, FMRIB Centre, University of Oxford. A Driven Equilibrium Single Pulse Observation of T_1_ (DESPOT1) sequence was used to ensure standardization of structural MRI scans across the 3 scanner platforms (Deoni et al. 2008; Ecker et al. 2012; Lai, Lombardo, Chakrabarti et al. 2012). In brief, 2 spoiled gradient recalled (SPGR) images were acquired at 2 flip angles (*α*) from which an estimate of the absolute *T*_1_ value was derived at each voxel. These quantitative *T*_1_ maps were used to create simulated *T*_1_-weighted inversion recovery (IR) images, with 176 contiguous slices (1 mm × 1 mm × 1 mm resolution), a field of view of 25.6 cm, a simulated repetition time/inversion time (TR/TI) of 1800/850 ms, a scaling constant *ρ* = 10 000, and a flip angle of 20°. This combination of parameters gave excellent deep and cortical GM/WM contrast for tissue segmentation without the need of modulation by *B*_0_ and *B*_1_ field inhomogeneities because compensation had been introduced during the estimation of absolute *T*_1_.

The simulated *T*_1_-weighted IR images were segmented and then registered to the standard Montreal Neurological Institute (MNI) space using SPM8 (http://www.fil.ion.ucl.ac.uk/spm). Native-space GM, WM, and cerebrospinal fluid (CSF) images were obtained using standard automated segmentation algorithm (Unified Segmentation). Total GM, WM, and CSF absolute volumes were estimated by summing up the partial volume estimate throughout each class of segment, and total brain volume (TBV) was estimated by summing up total GM and WM volumes. The native-space GM and WM images were then registered to a study-specific template generated from all ASC participants using a high-dimensional nonlinear diffeomorphic registration algorithm (DARTEL) (Ashburner 2007), with Jacobian modulation. The modulated standard-space images were then smoothed with a 4-mm full-width-half-maximum Gaussian kernel.

### Statistical Analysis

#### Neuroanatomical Correlates of a History of Language Delay: Voxel-wise Mass-Univariate Analysis

VBM was performed with SPM8, separately for GM and WM. To avoid edge effects between different tissue types, group comparisons were constrained to voxels in the study-specific template with a tissue probability >0.25. Prior to statistical modeling, each modulated GM or WM map was re-scaled (divided) by individual total GM or WM volume, resulting in maps indicative of “relative” regional GM or WM volume (i.e., regional GM/WM volume relative to individual total GM/WM volume). This individual-level adjustment (O'Brien et al. 2006) was done to avoid inadequate “control” for total volume differences in the general linear model (GLM) where total volume was included as a covariate but itself was significantly correlated with other independent variables (Miller and Chapman 2001). We used the binary variable of history of language delay as an independent variable and modeled age as continuous and scanning centers as categorical fixed-effect nuisance covariates (Suckling et al. 2012). Results were corrected for multiple comparisons by controlling the topological false discovery rate (FDR) at the cluster level, calculated under Gaussian Random Field Theory assumptions (Chumbley and Friston 2009), with a cluster-forming voxel-level height threshold of *P* < 0.025. Inference was constrained only to those clusters whose spatial extent exceeded the FDRc extent threshold [corrected for nonstationarity (Hayasaka et al. 2004)] that ensures a cluster-wise FDR at *q* < 0.05.

##### Region-of-interest approach to test the replicability of published VBM findings in the present sample

Since VBM analysis may suffer from insufficient power to detect small-effect group differences, we further tested the replicability of previous VBM findings (which themselves are various) using a region-of-interest (ROI) approach, which provides better power (type II error reduced). Four previous studies (Kwon et al. 2004; McAlonan et al. 2008, 2009; Toal et al. 2010) provided VBM results (in GM or WM) that could be used for ROI analysis. Peak coordinates of clusters showing significant volume (or density) differences between individuals with ASC with or without a history of language delay were extracted and transformed to MNI coordinates via Lancaster transform if initially given in the Talairach space. This in total gave 4 GM and 2 WM regions (see Table 3). A sphere ROI 6 mm in radius was built centered around each coordinate (using the MarsBaR toolbox for SPM); this size was chosen because it generated sphere ROIs generally comparable (or slightly smaller) in size with the clusters reported in these VBM studies, and this size of sphere ROI was commonly adopted in the literature employing similar analysis strategy. Average regional GM/WM volume for each individual was extracted for all ROIs. Group-wise comparisons on the relative (i.e., scaled by individual total volume) and absolute GM/WM volumes were done using independent sample *t*-tests.

##### Testing spatial similarity between 2 group-differences: ASC with language delay versus neurotypical, and ASC without language delay versus neurotypical

The main analyses above investigated neuroanatomical variations within ASC in relation to a history of language delay. To further understand the “commonality” between subgroups, we performed a subsidiary analysis to test the spatial similarity between 2 VBM group-difference maps: 1) ASC with language delay versus neurotypical, 2) ASC without language delay versus neurotypical.

A second DARTEL procedure was carried out with the 80 individuals with ASC [with (*n* = 38) or without (*n* = 42) a history of language delay] plus 57 neurotypical individuals not significantly different from either ASC subgroups in age (ASC with delay vs. neurotypical *P* = 0.051; ASC without delay vs. neurotypical *P* = 0.923) and full-scale IQ (FIQ) (ASC with delay vs. neurotypical *P* = 0.073; ASC without delay vs. neurotypical *P* = 0.758), using the same preprocessing pipeline as in the main analysis. VBM was carried out with the same statistical procedures, between ASC with language delay and neurotypical controls, and between ASC without language delay and neurotypical controls, respectively.

To examine the commonality between the 2 group-differences, we calculated spatial overlap between the 2 group-difference maps from voxel-level *P* < 0.05 down to *P* < 0.0001, to illustrate if the overlap pattern is consistent and to test whether it is significantly larger than that which occurred by random. The presence of nonrandom overlap indicates statistically significant commonality/similarity between the 2 group-differences, parallel to the disparity identified by the main VBM analysis directly comparing ASC individuals with and without language delay. We performed conjunction analyses with logical “AND” masking (Nichols et al. 2005) and computed the overlap as a proportion of the total number of suprathreshold voxels for each of the 2 maps (then took the average of the 2), repeatedly for 2 pairs of group-difference maps (“ASC with delay > neurotypical” AND “ASC without delay > neurotypical,” “neurotypical > ASC with delay” AND “neurotypical > ASC without delay”) from *P* = 0.05 to 0.0001 (incrementing at 0.0001). Here, we did not apply spatial extent thresholds because using a cluster-level FDR procedure to control for type I error will result in different spatial extent thresholds for different VBM comparisons, influencing the overlap analyses across group-difference maps; neither did we apply an arbitrary extent threshold as we were also examining how overlapping voxels were spatially distributed (i.e., contiguous versus dispersed). Additionally, examining across multiple voxel-level thresholds had already accounted for multiple comparison correction.

To test statistical significance, we ran Monte Carlo simulations (5000 iterations) to create the null distribution of random overlap at each voxel-level threshold from *P* = 0.05–0.0001 (500 in total, incrementing at 0.0001) to assess the probability that the overlap was not random (Lombardo et al. 2012; Lai, Lombardo, Suckling et al. 2013). For each simulation, we generated 2 whole-GM/WM maps filled with values sampled randomly from a Gaussian distribution and having the same spatial smoothness as the observed group-difference maps. The simulated maps were then thresholded at the same voxel-level threshold as the observed maps, and the percentage of overlapping voxels in the 2 suprathreshold simulated maps was calculated. Over the 5000 iterations, we constructed the null distribution of the overlap percentage that occurred by random. *P*-values were computed by counting the number of instances where overlap percentages were greater than or equal to the observed overlap percentage in the real data. Computations were performed with MATLAB version 2012b (The MathWorks, Inc., Natick, MA, USA).

#### Neuroanatomical Correlates of Current Language: PLS Analysis

To identify neural systems associated with a LV for current language ability, measured by the combination of VIQ, F-A-S, and NWR scores, we applied the multivariate statistical technique of PLS using PLSGUI (http://www.rotman-baycrest.on.ca/pls/). The goal of this “Behavioral PLS” is to take 2 multivariate matrices (one for behavioral variables and the other for brain variables) and find the combination of LVs from the brain and behavioral matrices that express the largest amount of common information (i.e., largest covariance) (McIntosh and Lobaugh 2004; Krishnan et al. 2011). This has been applied in studies of obsessive-compulsive disorder, autism, and psychotic disorder (Menzies et al. 2007; Ecker et al. 2012; Dean et al. 2013). In our case, this “1-group PLS” analysis identifies the set of brain voxels most correlated with the LV underlying the 3 current language measures in male adults with ASC.

Individually re-scaled GM and WM maps were standardized by age before entering PLS analysis. GM and WM images were used together, representing 2 “conditions” (McIntosh and Lobaugh 2004; Krishnan et al. 2011; Ecker et al. 2012). Analysis was also constrained to voxels in the study-specific template with a tissue probability >0.25. After PLS identified sets of correlated latent brain and behavioral variable pairs (LVs), statistical inferences from each of these LVs were tested with a permutation test (10 000 permutations).

To understand which voxels contribute most reliably to the latent brain–behavior variable pairs, bootstrapping (10 000 resamples) was implemented to derive estimates of the standard error for the “salience” at each voxel. A “bootstrap ratio” was computed by dividing each of the voxel “salience” by the standard error. This made the bootstrap ratio proportional to *z*-statistics, used for thresholding the results for visualization of voxels which most reliably contribute to the LVs; see Krishnan et al. (2011) for details. Here, we used a bootstrap ratio of 2.5 (∼*P* < 0.012) and a minimum cluster size of 400 voxels for visualization. The choice of these thresholds is arbitrary and is only for the purpose of visualization; it is not related to the main statistical inference made about the LVs via the permutation test.

##### Testing whether current language–neuroanatomy relationship is dependent on language developmental history

Lastly, in order to examine whether neural systems associated with current language are further dependent on whether there is a history of language delay, we carried out a second PLS analysis using exactly the same brain and behavioral (current language) measures as well as statistical procedures as in the above “1-group PLS” analysis, but this time with ASC individuals divided into 2 groups according to whether one had a history of language delay. This “2-group PLS” analysis aims at 1) testing whether there are significant LVs that account for the largest covariance of the data when both historical and current language measures are incorporated, and 2) testing whether current language–neuroanatomy relationship is dependent on the presence or absence of a history of language delay.

## Results

### Correlation Between a History of Language Delay and Behavioral Characteristics

Male adults with ASC with (*n* = 38) or without (*n* = 42) a history of language delay did not differ significantly in terms of age, performance IQ (PIQ), or FIQ (Table 1). They were not statistically different in terms of VIQ-PIQ discrepancy (*P* = 0.19). For current language, those without delay had significantly better word generativity with a medium effect size (*P* = 0.003, Cohen's *d* = 0.68), with trend-level higher VIQ (*P* = 0.07, *d* = 0.42), but performed comparably to those with delay on phonological memory (*P* = 0.52, *d* = 0.17).

**Table 1:** History of language delay and behavioral characteristics

*N* = 80	With delay (*N* = 38)	Without delay (*N* = 42)	Statistics		Effect size
	Mean (SD)	Mean (SD)	t/*U*	*P*	Cohen's *d*
Age	23.2 (5.6)	25.2 (5.6)	−1.63	0.11	0.36
FIQ	106.7 (13.1)	111.1 (15.7)	−1.36	0.18	0.30
PIQ	106.1 (14.2)	108.0 (16.5)	−0.54	0.59	0.12
VIQ	105.6 (12.6)	111.6 (15.7)	−1.86	0.07	0.42
FAS^a^	33.8 (11.8)	41.3 (10.3)	−3.02	0.003	0.68
NWR^b^	21.1 (4.5)	21.8 (3.9)	−0.65	0.52	0.17
ADIR-S	20.7 (5.1)	17.0 (4.9)	3.29	0.001	0.74
ADIR-C	15.0 (4.2)	13.5 (3.7)	1.72	0.09	0.38
ADIR-RRB^c^	5.0 (4.0)^c^	5.0 (3.0)^c^	*786*^c^	0.91	0.03^d^
ADOS-SC^e^	10.5 (5.2)	8.6 (4.0)	1.82	0.07	0.41
ADOS-RRB^c,e^	1.0 (2.0)^c^	1.0 (2.0)^c^	*665*^c^	0.43	0.18^d^
AQ	30.1 (8.0)	28.8 (9.3)	0.67	0.51	0.15
EQ	23.5 (13.1)	25.4 (11.7)	−0.69	0.49	0.15
Eyes Test^f^	21.3 (5.6)	22.8 (5.6)	−1.11	0.27	0.27
VIQ-PIQ diff	−0.5 (12.9)	3.6 (14.8)	−1.31	0.19	0.29

Note: SD, standard deviation; FIQ, full-scale IQ; PIQ, performance IQ; VIQ, verbal IQ; FAS, word generativity “F-A-S” task; NWR, non-word repetition task; ADI-R, Autism Diagnostic Interview-Revised; ADIR-S, ADI-R diagnostic algorithm social reciprocity subscore; ADIR-C, ADI-R diagnostic algorithm communication subscore; ADIR-RRB, ADI-R diagnostic algorithm restricted and repetitive behaviors subscore; ADOS, Autism Diagnostic Observation Schedule; ADOS-SC, ADOS diagnostic algorithm social-communication subscore; ADOS-RRB, ADOS diagnostic algorithm restricted and repetitive behaviors subscore; AQ, Autism Spectrum Quotient; EQ, Empathy Quotient; Eyes Test, accuracy on the “Reading the Mind in the Eyes” Test; VIQ-PIQ diff, discrepancy between VIQ and PIQ.

^a^Data available for 78 participants.

^b^Data available for 77 participants.

^c^Distribution significantly deviant from normality so nonparametric Mann–Whitney *U*-test was performed; median and interquartile range are provided instead of mean and standard deviation.

^d^Equivalent Cohen's *d* calculated from Pearson's *r*.

^e^Data available for 77 participants.

^f^Data available for 77 participants.

In terms of autistic features, those with delay had more childhood social reciprocity difficulties on the ADI-R (*P* = 0.001) with a medium to large effect size (*d* = 0.74), and a trend-level higher current ADOS social-communication symptom score (*P* = 0.07, *d* = 0.41). The 2 groups, however, showed no significant differences on childhood ADI-R communication (verbal) and repetitive and restricted behaviors (RRB) scores, current RRB on the ADOS, advanced mentalizing ability, or self-reported autistic and empathy traits.

### Correlation Between Current Language Ability and Behavioral Characteristics

**Figure 1.**
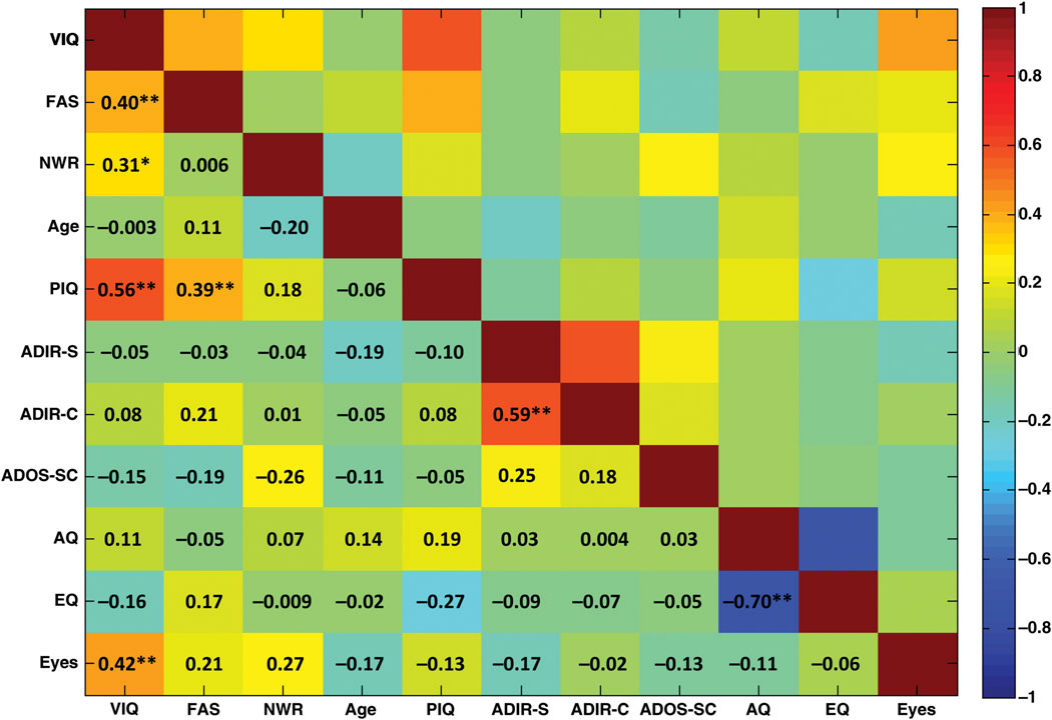
Current structural language abilities and behavioral characteristics. This correlation matrix shows the pair-wise Pearson's correlations among current language measures and demographic/behavioral characteristics. Color-coding indicates the strength of the correlation, and each cell gives the Pearson's *r*. *P < 0.01, **P < 0.001.

Within the 3 measures of current language, VIQ was significantly positively correlated with both F-A-S and NWR scores, with the latter 2 not significantly correlated (Fig. 1). This pattern supports our rationale for a multivariate imaging approach (PLS) because the positive correlations suggest latent factors underlying all language variables.

None of the behavioral measures correlated with age. PIQ positively correlated with VIQ and F-A-S score. None of the current language measures correlated with autistic features, except for a significant positive correlation between VIQ and accuracy on the Eyes Test, a finding also reported by others (Peterson and Miller 2012). Among measures of autistic features, as expected, ADI-R social reciprocity and communication subscores significantly positively correlated with each other, and AQ significantly negatively correlated with EQ.

### Neuroanatomical Correlates of a History of Language Delay

**Figure 2.**
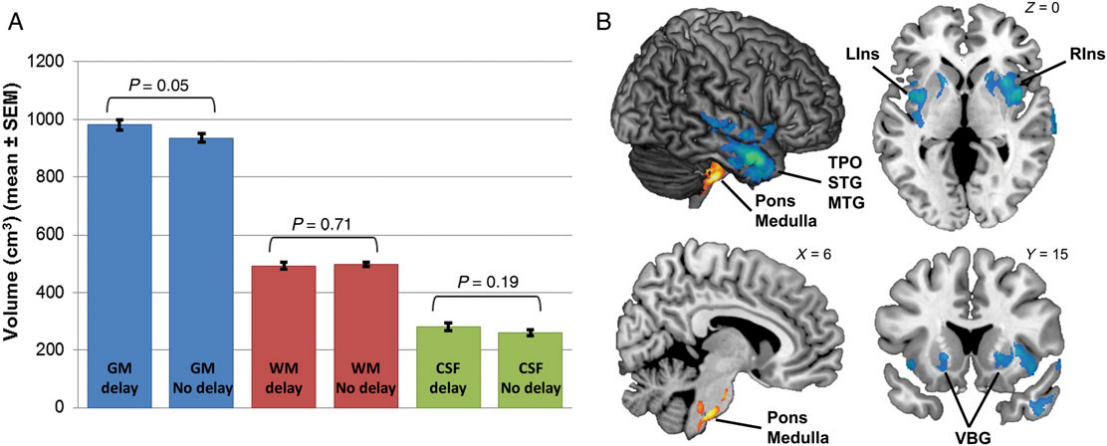
Neuroanatomical correlates of history of language development in ASC. (*A*) Bar graphs illustrate absolute total GM, WM, and CSF volume differences between ASC individuals with and without a history of language delay. Those with delay showed significantly larger total GM volume than those without. Error bar represents standard error of the mean (SEM). (*B*) Regions where relative GM volume differed between those with and without a history of language delay. Blue/green regions depict areas where relative regional GM volume was decreased in those with a history of language delay compared with those without; orange/yellow regions depict areas where relative regional GM volume was increased in those with a history of language delay compared with those without. LIns, left insula; Medulla, medulla oblongata; MTG, middle temporal gyrus; RIns, right insula; STG, superior temporal gyrus; TPO, temporal pole; VBG, ventral basal ganglia.

Those with a history of language delay had a significantly (*t*_(78)_ = 1.99, *P* = 0.05) larger absolute total GM volume (mean ± standard deviation: 980 ± 111 cm^3^) than those without delay (934 ± 96 cm^3^) (Fig. 2A). The 2 groups showed no significant differences on absolute total WM (with delay: 492 ± 72 cm^3^; without delay: 498 ± 54 cm^3^; *t*_(78)_ = −0.38, *P* = 0.71) and CSF volumes (with delay: 282 ± 83 cm^3^; without delay: 259 ± 69 cm^3^; *t*_(78)_ = 1.32, *P* = 0.19).

At a regional level (Fig. 2B), VBM identified 3 GM clusters that were significantly smaller in those with compared with those without language delay, in terms of “relative” regional volume. Two of these clusters were located bilaterally in the insula and ventral basal ganglia (left-lateralized cluster size *k*_e_ = 6,232 voxels, cluster-level FDR-corrected *q* = 0.001, peak-voxel MNI coordinate [−32, −4, 8], *T* = 4.74; right-lateralized cluster *k*_e_ = 8,875 voxels, cluster-level *q* < 0.001, peak-voxel [40, 0, 7] *T* = 5.01), while the third cluster was located in right temporal pole, superior and middle temporal gyri (STG, MTG), and superior temporal sulcus (*k*_e_ = 7,325 voxels, cluster-level *q* < 0.001, peak-voxel [60, 5, −27] *T* = 5.79). On the other hand, one cluster located within the pons and medulla oblongata (*k*_e_ = 4,777 voxels, cluster-level *q* = 0.013, peak-voxel [8, −27, −46] *T* = 4.64) was significantly larger in those with compared with those without language delay. VBM on WM showed no regions that were significantly different in volume between those with or without language delay.

From these findings, perhaps the most surprising is that canonical language-related structures (e.g., Broca's and Wernicke's areas, other left-lateralized frontal, temporal, and inferior parietal regions) were not associated with a history of language delay. Since power issue may have limited the ability to detect small-effect sizes in these canonical language-related areas, we additionally performed ROI analyses on 13 canonical language-related ROIs defined from functional neuroimaging studies (http://web.mit.edu/evelina9/www/funcloc/funcloc_parcels.html) (Fedorenko et al. 2010). Group differences on relative GM volumes of these 13 ROIs were assessed by multivariate analysis of covariance (MANCOVA), where age and centers were included as nuisance covariates as in the VBM analysis. This analysis showed that individuals with or without language delay did not differ in relative GM volume in canonical language regions (Hotelling's Trace = 0.158, *F*_13,63_ = 0.766, *P* = 0.69; see Table 2 for post hoc ANCOVA for each ROI).

**Table 2:** Lack of volumetric differences in canonical language regions between ASC individuals with and without a history of language delay: post hoc ANCOVAs (after MANCOVA)

Region of interest	*F*_1,75_	*P*-value
Left angular gyrus	0.374	0.542
Left anterior temporal lobe	0.404	0.527
Left cerebellum	0.100	0.753
Left inferior frontal gyrus	0.648	0.423
Left orbital inferior frontal gyrus	0.035	0.852
Left middle frontal gyrus	1.089	0.300
Left middle-anterior temporal lobe	0.110	0.741
Left middle-posterior temporal lobe	1.127	0.292
Left posterior temporal lobe	0.200	0.656
Left superior frontal gyrus	2.646	0.108
Right cerebellum	0.015	0.902
Right middle-anterior temporal lobe	4.328	0.041
Right middle-posterior temporal lobe	0.129	0.720

Finally, the ROI approach testing for replicability of previous VBM findings in the present sample (Table 3) showed that most previous findings could not be replicated here. One exception is that there was a smaller relative GM volume in ASC individuals with language delay compared with those without, in the ROI at thalamus and basal ganglia reported by McAlonan et al. (2008).

**Table 3:** Region-of-interest (ROI) analysis (based on previous VBM studies) for volumetric differences between ASC individuals with (“D,” HFA) and without (“nD,” AS) a history of language delay

ROI index	ROI [MNI coordinate]^a^	Previous report	Present finding (relative volume)^b^	Present finding (absolute volume)
*GM*				
Kwon (1)	Middle cingulate gyrus [10, 1, 38]	D > nD	D ≈ nD (*t*_78_ = −0.40, *P* = 0.69)	D ≈ nD (*t*_78_ = 1.29, *P* = 0.20)
McAlonan (1)	Thalamus and basal ganglia [−18, −8, −0]	D < nD	D < nD (*t*_78_ = −2.06, *P* = 0.04)	D ≈ nD (*t*_78_ = −0.40, *P* = 0.69)
McAlonan (2)	Posterior cingulate and precuneus [−1, −50, 45]	D < nD	D ≈ nD (*t*_78_ = 0.45, *P* = 0.65)	D ≈ nD (*t*_78_ = 1.66, *P* = 0.10)
Toal (1)	Superior temporal gyrus and inferior parietal lobule [62, −20, 11]	D > nD	D ≈ nD (*t*_78_ = 0.04, *P* = 0.97)	D ≈ nD (*t*_78_ = 1.20, *P* = 0.24)
*WM*				
McAlonan (1)	Internal capsule [−14, −17, −9]	D > nD	D ≈ nD (*t*_78_ = 0.04, *P* = 0.97)	D ≈ nD (*t*_78_ = −0.35, *P* = 0.73)
Toal (1)	Beneath medial prefrontal cortex [−16, 46, 21]	D < nD	D ≈ nD (*t*_78_ = 0.66, *P* = 0.51)	D ≈ nD (*t*_78_ = 0.10, *P* = 0.92)

^a^When Talairach coordinates were provided in the initial reports, they were transformed into MNI coordinates (to be compatible with other concurrent analyses) using Lancaster transform.

^b^Adjusted/scaled by individual total GM/WM volume.

### Spatial Commonality Between “ASC with Language Delay versus Neurotypical” and “ASC without Language Delay versus Neurotypical” VBM Group-Difference Maps

**Figure 3.**
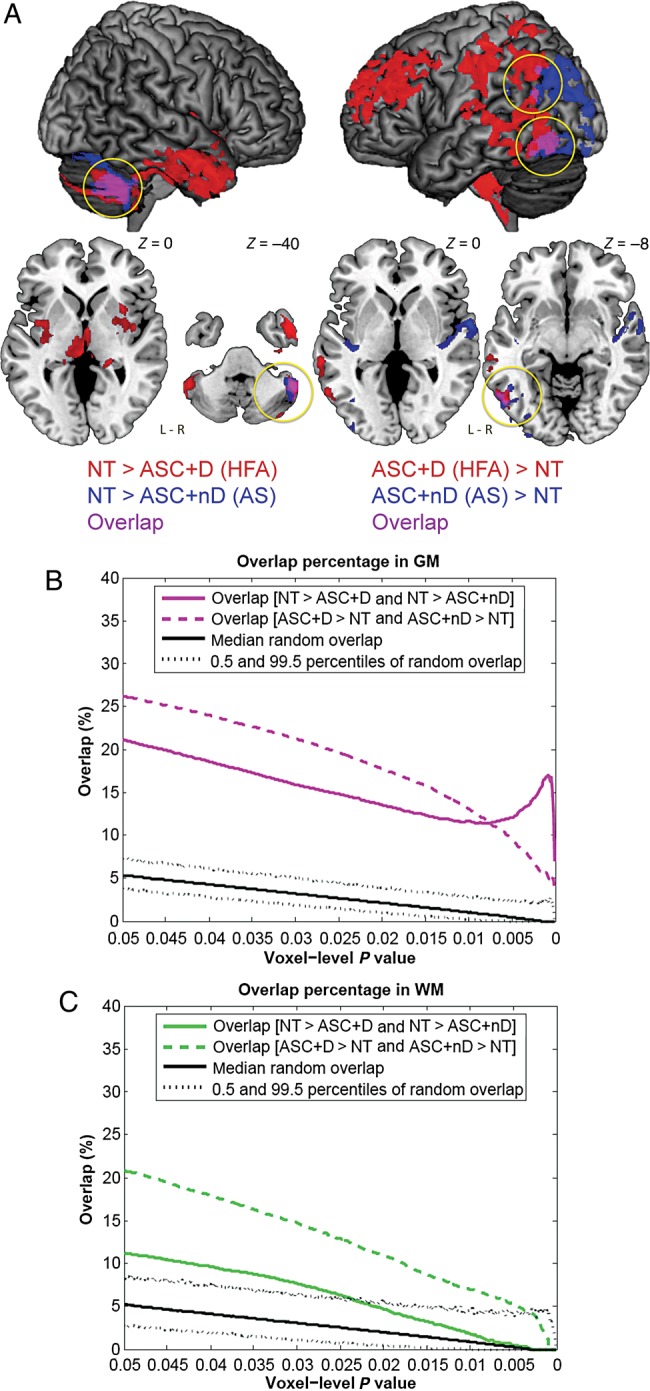
Commonality in neuroanatomy between ASC with versus without language delay when, respectively, contrasted to a neurotypical group. (*A*) On a selected threshold (voxel-level *P* < 0.025, cluster-level topological FDR *q* < 0.05), GM VBM group-difference maps of neurotypical (NT) versus ASC with language delay (ASC+D) and that of NT versus ASC without language delay (ASC+nD) overlapped (purple, also marked by yellow circles) at right cerebellum, left temporo-parietal junction and inferior posterior temporal region. (*B*) Across voxel-level thresholds, in GM NT–ASC+D and NT–ASC+nD group differences consistently showed nonrandom (i.e., larger than random condition) spatial overlap, indicating statistically significant commonality. Disparity between the two, however, was also present. Black solid line indicates the median overlap occurred under random condition derived from 5000 Monte Carlo simulations, with dotted lines indicating the 0.5 and 99.5 percentiles of the null distribution. (*C*) Across voxel-level thresholds, in WM NT–ASC+D and NT–ASC+nD group differences showed nonrandom spatial overlap but only in the less stringent thresholds.

Under the same statistical threshold as in the main VBM analysis above, here the additional VBM showed (Supplementary Table 1) that the neurotypical group had larger relative regional GM volume than the ASC with language delay (ASC+D) group in 6 clusters (involving bilateral cerebellum, thalamus, putamen, amygdala, hippocampus, insula, and right anterior temporal lobe); also the neurotypical group had larger relative regional GM volume than the ASC without language delay (ASC+nD) group in 1 cluster (at right cerebellum). These 2 group-difference maps overlapped mainly at right cerebellum (Fig. 3*A*, left). Additionally, the neurotypical group had smaller relative regional GM volume than the ASC+D group in 7 clusters (involving bilateral prefrontal and parietal cortices, temporo-parietal junction, precuneus, pons and medulla, and left posterior temporal cortex); also the neurotypical group had smaller relative regional GM volume than the ASC+nD group in 4 clusters (involving bilateral Heschl and superior temporal gyri, and left temporo-parieto-occipital junction). These 2 group-difference maps overlapped mainly at left temporo-parietal junction and inferior posterior temporal region (Fig. 3*A*, right). For WM, only the comparison between neurotypical and ASC+D groups survived statistical control, which found that the neurotypical group was smaller in 1 cluster involving right posterior frontal and anterior parietal regions.

It is important to note that although overall the “spatial extent” of deviation from the neurotypical group seems greater in the ASC+D than in the ASC+nD group (under the present threshold), one cannot infer where in the brain that shows statistically significant ASC+D versus ASC+nD differences in “magnitude (volume)” by visually comparing the “ASC+D versus neurotypical” and “ASC+nD versus neurotypical” group-difference maps. Localization of these volumetric group differences can only be statistically adequately revealed by directly comparing the ASC+D and ASC+nD groups, as already shown by the main VBM analysis (Fig. 2*B*) (Nieuwenhuis et al. 2011).

Some of these findings in subgroups (e.g., neurotypical > ASC+nD and ASC+D at cerebellum; neurotypical < ASC+D at prefrontal and parietal cortices, and neurotypical < ASC+nD at Heschl and superior temporal gyri) were comparable with the neurotypical-ASC (whole-group) differences reported earlier from a larger cohort including the present samples (Ecker et al. 2012), despite using different sample sizes, preprocessing pipelines, and statistical models (in this study: parametric GLM, age covaried, and corrected for total volume at the individual level; in the earlier larger sample study: nonparametric GLM, age not covaried, and corrected for total volume at the model level, and PLS was additionally used). More importantly, the overlap at left inferior posterior temporal region replicated findings from meta-analysis that the same region is larger in both ASC+D and ASC+nD subgroups when contrasting with neurotypical groups (Yu et al. 2011).

Across voxel-level thresholds, the group-difference map between neurotypical and ASC+D groups, and that between neurotypical and ASC+nD groups, consistently showed nonrandom spatial overlap in GM in both directions of contrasts (Fig. 3*B*). The extent of overlap was about 10–20% depending on the voxel-level threshold. This indicates that there is significant, nonrandom spatial commonality between the two, yet they are still not quite the same. In WM, such overlap was present but was less consistent, that they became nonsignificant at the more stringent *P*-values (Fig. 3*C*).

### Neuroanatomical Correlates of Current Structural Language

**Figure 4.**
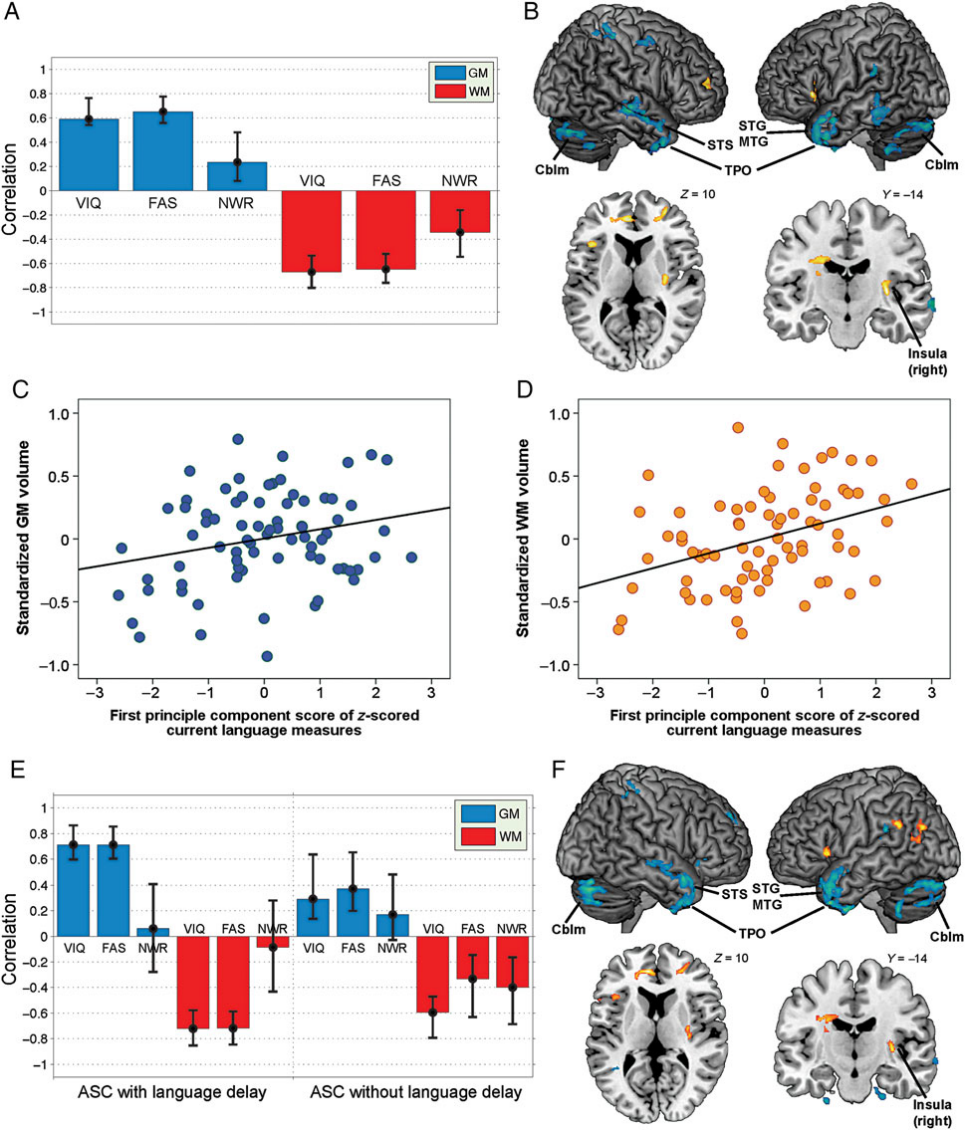
Neuroanatomical correlates of current structural language in ASC. (*A*) This “correlation overview” graph shows that for the significant LV pair (LV1) revealed by the 1-group PLS analysis, there were stable correlations (i.e., confidence intervals not including zero; error bar representing bootstrap-estimated 95% confidence interval) between the “brain scores” (i.e., the dot-product of the brain LV saliences and the individual's imaging data, giving an overall summary of the brain data for each individual) (McIntosh and Lobaugh 2004) and all 3 language measures. This also illustrates that overall the correlation between the “brain scores” and language performance was contributed by opposite directions of correlation for the GM and WM “conditions.” (*B*) Different sets of brain regions, altogether, contribute to LV1 in the 1-group PLS analysis, visualized at the thresholds of voxels with a ︱bootstrap ratio︱> 2.5 and clusters larger than 400 voxels. Blue/green regions (mostly in GM) show the most reliable voxels with “positive” saliences (bootstrap ratios), where language performance was positively correlated with a higher probability being GM than WM voxels, suggestive of larger GM volume; orange/yellow regions (mostly in WM) show the most reliable voxels with “negative” saliences, where language performance was positively correlated with a higher probability being WM than GM voxels, suggestive of larger WM volume. (*C* and *D*) Scatter plots conceptually illustrate the relationships described in (*B*) for the sets of brain regions contributing to LV1. Panel (*C*) demonstrates the positive correlation between current language ability and age-standardized relative GM volume for brain regions with positive saliences; panel (*D*) demonstrates the positive correlation between current language ability and age-standardized relative WM volume for brain regions with negative saliences. *X*-axis indicates the first principle component score of *z*-scored language measures, a summary index for overall current language ability. *Y*-axis in (*C*) indicates the average age-standardized relative GM volume from the blue/green regions in (*B*), and in (*D*), the average age-standardized relative WM volume from the orange/yellow regions in (*B*). Panels (*E*) and (*F*), parallel to (*A*) and (*B*), show the correlation overview and neural systems in the significant LV pair (LV1) revealed by the 2-group PLS analysis. (*E*) demonstrates that the brain–behavior correlation patterns were overall similar between those with and without a history of language delay. (*F*) shows the spatial involvement of LV1 from the 2-group PLS analysis [visualized at the same thresholds as in (B)], which involves mostly the same structures as those of the LV1 from the 1-group PLS analysis. Cblm, cerebellum; MTG, middle temporal gyrus; STG, superior temporal gyrus; STS, superior temporal sulcus; TPO, temporal pole.

Only participants without any missing data in the current language measures (*n* = 76; with language delay *n* = 35, without language delay *n* = 41) were included in these analyses (Fig. 4). None of the 3 language measures showed a significant correlation with total GM, WM, or CSF volumes, except a small positive correlation between VIQ and total GM volume (*r* = 0.24, *P* = 0.03). Across all ASC participants, the 1-group PLS analysis identified one (marginally) significant LV pair (LV1, singular value = 207.42, *P* = 0.052) using the permutation test, which accounted for 29.95% of covariance between regional brain volume and current language; all other 5 LV pairs were nonsignificant (LV2 *P* = 0.766; LV3 *P* = 0.491; LV4 *P* = 0.999; LV5 *P* = 0.168; LV6 *P* = 0.358). This significant LV pair included a set of brain regions (mostly in GM) for which current language was positively correlated with a higher probability being GM than WM voxels (suggestive of larger relative regional GM volume), involving mainly (but not exclusively) bilateral temporal pole, STG and MTG, superior temporal sulci (STS), cerebellum, left inferior parietal lobule, and right dorsolateral fronto-parietal regions. It also included a set of regions (mostly in WM) for which current language was positively correlated with a higher probability being WM than GM voxels (suggestive of larger relative regional WM volume), involving mainly bilateral prefrontal regions partially overlapping with the frontal portion of cingulum extending into left anterior cingulate gyrus, corticospinal tract at the level above caudate, and WM adjacent to right insula. See Figure 4*B*.

Finally, the 2-group PLS analysis identified one significant LV pair (LV1, singular value = 308.55, *P* = 0.025) which accounted for 16.79% of covariance between regional brain volume and current language, when language developmental history was also taken into account; all other 11 LV pairs were nonsignificant (LV2 *P* = 0.170; LV3 *P* = 0.237; LV4 *P* = 0.851; LV5 *P* = 0.300; LV6 *P* = 0.585; LV7 *P* = 0.994; LV8 *P* = 1.000; LV9 *P* = 0.575; LV10 *P* = 0.598; LV11 *P* = 0.866; LV12 *P* = 0.999). This significant LV pair involved mostly the same anatomical structures as revealed by the previous 1-group PLS analysis (Fig. 4*F*). Critically, the brain–behavior correlation patterns were similar for both subgroups (Fig. 4*E*), which were also comparable with that revealed across all ASC individuals in the 1-group PLS analysis (Fig. 4*A*). This indicates that current language–neuroanatomy relationship is similar across ASC subgroups with or without a history of language delay (i.e., not dependent on language developmental history).

## Discussion

This study investigated the neural correlates of individual differences in language, in high-functioning male adults on the autism spectrum. Specifically, we assessed if a history of language delay and current structural language abilities are related to neuroanatomical variations. A history of language delay predicted adulthood deficits on word generativity and verbal IQ. Individuals with a history of language delay on average also had a subtle increase in absolute total GM volume, compared with those without a delay. More localized in neuroanatomy, a history of language delay was associated with smaller relative regional volume (i.e., adjusted for individual total volume) at insula, ventral basal ganglia, superior/middle and polar temporal regions, and larger relative regional volume at pons and medulla oblongata. These findings suggest that individuals with ASC, differentiated simply by the presence or absence of early language delay, are different in terms of adulthood current language function, and specific aspects of neuroanatomy. In adulthood, better current language was associated with increased relative GM volume in distributed regions mainly (but not exclusively) in bilateral temporal pole, superior and middle temporal regions, and dorsolateral fronto-parietal and cerebellar structures, as well as increased relative WM volume in distributed frontal and insular regions. Additionally, current language–neuroanatomy correlation patterns were similar across ASC subgroups with or without a history of language delay, marking aspects of commonality across ASC subgroups by language onset.

The findings are important for 2 reasons. First, the results suggest that both current language in adulthood and early language development contribute to heterogeneity within the autism spectrum, at both the neural and cognitive levels. Substantial heterogeneity is a limiting factor impeding research progress in understanding “the autisms” (Waterhouse 2013), so one way to reduce heterogeneity may be by taking into account both language developmental history and current language functioning.

Second, the results are important for how research should proceed following the publication of DSM-5, which may have changed how we conceptualize factors that are “additional” to the core diagnosis of ASD (as clinical specifiers, and/or as co-occurring diagnoses). The main reason behind why DSM-5 rejects subtypes and eschews variables such as early language delay is because of the seemingly arbitrary nature of early developmental cutoffs for stating that an individual has language delay or not, so as to increase the reliability of clinical diagnosis. However, the concerns of the validity and reliability of historical information in clinical practice should not be taken to mean that variation in early language history per se is not important. Individual differences in language within the autism spectrum will still help delineate key developmental mechanisms.

### History of Language Delay is Associated with Adulthood Structural Language Ability and Neuroanatomy

Previous efforts to identify how language delay has an impact on later behavior and cognition in individuals with ASC have been inconclusive, possibly due to inconsistent diagnostic definitions (of “AS” vs. “HFA”) and circularity in research design (Witwer and Lecavalier 2008). Nevertheless, it has been repeatedly shown that those without language delay tend to show fewer autistic symptoms in social communication in childhood (Szatmari et al. 1995; Ozonoff et al. 2000; Verte et al. 2006), and that this difference dissipates with age (Howlin 2003; Witwer and Lecavalier 2008). Individuals without language delay may also have greater social motivation and better ability to engage in prosocial behavior (Macintosh and Dissanayake 2004). Our findings are largely consistent with these: Those without language delay had fewer childhood difficulties in social reciprocity (measured by ADI-R) but showed mostly comparable current social communication characteristics (measured by ADOS, AQ, EQ, Eyes Test) as those with delay, suggesting a possible “catch-up” in the language-delayed subgroup, as also found in childhood studies (Bennett et al. 2014). On the other hand, we also found that early language delay was associated with poorer adulthood word generativity and VIQ (marginally), corroborating findings showing similar associations in childhood (Mayo et al. 2013). Overall these indicate potentially long-lasting effects of delayed language onset on specific aspects of cognitive development in autism.

In terms of neuroanatomy, we observed greater absolute total GM volume on average in men with a history of language delay compared with those without, a pattern comparable with that observed in boys (Lotspeich et al. 2004). An increase in TBV in young children with autism is found in males with a history of “regression” but not in other subgroups (e.g., males without regression, or females) (Nordahl et al. 2011). Whether the “early language delay–larger current GM” association is neurodevelopmentally related to the “regression–early brain overgrowth” association awaits investigation. We might speculate that greater GM may reflect excessive neurogenesis or reduced apoptosis in early development, resulting in increased local neural connectivity and decreased signal-to-noise ratio (Belmonte et al. 2004; Courchesne et al. 2007) underlying language developmental difficulties. Such hypotheses await neuropathological studies in relation to language delay in ASC.

As adults, those with delay had significantly smaller relative regional GM volume in bilateral insula and ventral basal ganglia, and right temporal pole, STG, MTG, and STS than those without delay, despite the 2 groups being comparable on current social-emotional processing (measured by the EQ and the Eyes Test) and autistic features (measured by the ADOS and AQ). ROI analysis further suggests that a history of language delay does not have significant long-lasting effects into adulthood on “canonical language circuitry.” However, the insula, temporal pole, and superior temporal gyrus/sulcus are in some studies still important structures involved in language processing (Price 2010). For example, patients with semantic dementia have atrophy in anterior temporal pole and superior temporal regions (Patterson et al. 2007); lesion mapping studies have implicated the insula in verbal fluency (Bates et al. 2003); and functional MRI studies have identified insula as involved in sentence comprehension, possibly related to affective perspective-taking (Basnakova et al. 2014). Evidence linking anterior/superior temporal atrophy and insular lesions to semantic/fluency deficits, in conjunction with our results of deficits in fluency and semantic concept retrieval in individuals with a history of language delay, suggest a substantial impact of developmental language delay on current language processing in autism. Although canonical language-related regions were not affected, we caution against the interpretation that such delay does not have a long-lasting neurobiological impact. Notably, insula and anterior/superior temporal regions were associated with a history of language delay and both have strong links to functional language deficits similar to those seen in this study.

We also observed larger relative regional GM volume in pons and medulla oblongata in those with language delay. These brainstem structures are typically involved in modulating basic physiology and behavior (e.g., the monoamine systems, and sensory, motor, autonomic, reflexive responses) (Clark et al. 2010), so it is difficult to specifically relate language delay in autism with these. One interpretation of this unexpected association is that the observed volumetric difference is simply a marker for other factors associated with language developmental delay that are not necessarily implicated in language processing per se.

The present results may be helpful for generating new hypotheses about the cognitive and neurobiological implications of early language delay in autism. First, given the links between ventral basal ganglia and insula in reward/statistical learning and general salience processing (Menon and Uddin 2010; Diekhof et al. 2012), our findings suggest that language delay in autism may signal deficits or atypical processing in these domains. The earlier finding that individuals with ASC and language delay may be less socially motivated than those without (Macintosh and Dissanayake 2004) fits well with this hypothesis. The insula is a key component processing salience, and it is also a hub modulating and switching between different intrinsic functional networks (Menon and Uddin 2010), having a high base-rate for being active regardless the sort of cognitive tasks (Yarkoni et al. 2011). Ventral basal ganglia is involved broadly in motivation and learning (Graybiel 1995; Bjorklund and Dunnett 2007). Taken together, they subserve information/salience processing and learning in a domain-general manner. This observation is consistent with the possibility that language delay in autism might be related to atypical attribution of salience to social stimuli and/or reward learning (Scott-Van Zeeland et al. 2010; Sims et al. 2012; Heerey 2014).

Second, given that social deficits are linked to communication/language deficits in autism, it may be that language delay in autism is a marker for atypical social development. Thus, delayed language in autism may be better explained as reflecting (or even originating from) more pronounced impairments in dyadic relations which are foundational for early social-communicative development (Baron-Cohen 1995; Boucher 2012). Regions identified here smaller in individuals with a history of language delay all play critical roles in social-communicative development. The insula critically involves in affective processing (Adolphs 2009), introspective awareness (Critchley et al. 2004), and empathy (Singer et al. 2004). The ventral basal ganglia are strongly innervated by the midbrain dopaminergic system, and responds to social and nonsocial rewards (Haber and Knutson 2010). Superior temporal gyrus/sulcus is implicated in processing social-perceptual cues that paramount in early development such as eye gaze, biological motion, and processing of actions (Pelphrey et al. 2011). One hypothesis that our observations lead to is that language delay might be a residual effect of a less well-developed neural system underpinning processing of social-communicative cues. The transactional nature of language and social-communicative development in autism highlights the need for longitudinal investigations (Baron-Cohen et al. 1997; Charman et al. 2000).

The VBM findings we observed here largely do not replicate previous reports from smaller samples in different age groups (i.e., children and adolescents) (Kwon et al. 2004; McAlonan et al. 2008, 2009). This lack of replication may reflect heterogeneity associated with age and development that substantially affects the neuroanatomy of autism (Duerden et al. 2012). Our findings also do not replicate a previous report of a smaller sample of adults (Toal et al. 2010), which was different from ours in terms of demographic characteristics: it included females, older adults (up to 59 years), and individuals with below-average IQ (down to 53). To confirm if we could replicate these findings with better-powered analyses, we performed ROI analyses based on regions showing significant group differences in these previous VBM studies. However, even this approach could not replicate most earlier findings in the present dataset, apart from the one at thalamus and basal ganglia (McAlonan et al. 2008). The substantial sample differences across studies in age, sex, IQ, participant number, and differences in analysis pipeline most likely all contribute to heterogeneity and nonoverlapping results.

### Current Language is Associated with Brain Regions Underpinning Executive, Language, and Social Processing

Functional MRI studies have found differential neural activation patterns during language tasks between males with and without ASC. For example, during verbal fluency tasks, those with ASC show decreased activation at premotor cortex, anterior cingulate, insula, putamen, and fusiform gyrus (Kenworthy et al. 2013), and increased activation at right inferior frontal and superior temporal cortices (along with reduced hemispheric lateralization) (Kleinhans et al. 2008). During semantic processing tasks, individuals with ASC show reduced activation at left inferior frontal cortex (Harris et al. 2006; Lo et al. 2013) but increased activation at left posterior superior temporal cortex (Harris et al. 2006). A mixture of hypo- and hyperactivation at canonical language and executive function regions marks potential functional neural characteristics of autism at the group level when compared with neurotypical individuals. Our study further extends the investigation into individual differences within the autism spectrum, at the structural level.

Current language measures here cover word knowledge, simple semantic processing (i.e., concept formation and verbal reasoning) and language-related executive control (i.e., phonological working memory, word generativity, and verbal reasoning), so it is unsurprising to find an association with GM systems involved in word processing and semantic knowledge (e.g., STG, STS, MTG, temporal pole, cerebellum) (Fedorenko et al. 2010; Friederici 2012), as well as in executive control (e.g., fronto-parietal structures, cerebellum) (Barbey et al. 2012; O'Halloran et al. 2012). Importantly, these structures also underpin key components of social processing: the temporal pole is known to integrate social knowledge (Frith 2007; Olson et al. 2013) and the superior temporal structures code for biological motion and visual social perception (Allison et al. 2000; Nummenmaa and Calder 2009). In addition, the WM system associated with current language is adjacent to regions associated with social-emotional processing (e.g., cingulum and WM beneath insula). These may mark the closely entwined and shared neurobiological substrates between language and social processes. For example, the fact that developmentally language and social processing share common neural substrates (e.g., the STS) indicates their close linkage in acquisition (Redcay 2008). Our results demonstrate that in high-functioning male adults with ASC, neuroanatomy does vary as a function of structural language ability, but this should be interpreted in light of the well-established close relationship between language and social processes (Baron-Cohen et al. 1997).

### Commonality and Heterogeneity Within the Autism Spectrum

Although we showed that within the umbrella ASC category, variations in current and historical language were associated with differences in neuroanatomy, such variability should not be interpreted as implying that there are completely distinct subgroups. When characterized by contrasting with neurotypical adult males, the neuroanatomy of adult males with ASC with or without a history of language delay showed nonrandom spatial overlap. This statistically significant overlap implies that although in ASC neuroanatomy varies with language development, subgroups still share substantial commonality. Nevertheless, the spatial overlap, though significantly different from random, is not extensive (∼10–20% in GM), indicating that commonality exists in parallel with disparity, as also shown by a meta-analysis (Yu et al. 2011).

Such commonality across the umbrella ASC category is further corroborated by the 2-group PLS findings that current language–neuroanatomy relationships were consistent irrespective of a history of language delay. This suggests that how current language ability is associated with current neuroanatomy is not determined upon whether one has a delayed language onset, indicating an important aspect of commonality across the autism spectrum. On the other hand, it is equally worth noting that despite the similarity of overall brain–behavior correlation patterns across subgroups with or without language delay, there seemed to be trend-level quantitative differences: The strength of the correlations between brain volume and VIQ and F-A-S scores was marginally stronger (and less variable) in the delayed than in the no-delay subgroups. We may hence speculate that the underlying neurobiology of those with language delay is more clear-cut, owing to the better mapping of brain volume onto current language variations; those without language delay, on the other hand, may be more varied themselves as a subgroup. The co-existence of the overall similarity in brain–behavior correlation patterns, and the trend-level differences in the strength of correlations contributing to aspects of the overall pattern, once again marks the importance of acknowledging both commonality and heterogeneity within the autism spectrum.

These neuroanatomical findings correspond well with recent electrophysiological findings in children, showing that within a neurotypical–ASC dichotomy, clinically diagnosed AS falls closer to ASC than to neurotypical, yet when compared directly with other diagnoses in ASC, AS is distinctly separate (Duffy et al. 2013). We suggest that appreciating commonality is as important as recognizing differences when it comes to clarifying the heterogeneity within ASC. We need to move away from the “forced choice” of conceptualizing ASC as either a unitary category or a collection of distinct subgroups. Our findings confirm that both commonality and heterogeneity in ASC are evident. A better understanding requires sufficient appreciation of both.

### Limitations and Prospects

Several limitations must be acknowledged. First, a history of language delay was assessed via parental recollection and thus all caveats (e.g., loss of detailed information, memory bias influenced by current perception, etc.) that are associated with retrospective reports apply. Such limitations can only be avoided by using a prospective longitudinal design. Retrospective parental recall may suffer from a “telescoping” bias, especially for language milestones. That is, the older the individual is when the parents are interviewed, the later the month of language onset is reported to have been (Hus et al. 2011). However, this effect is more prominent for parents with children who have low verbal IQ. Our participants are individuals without intellectual disability and with an average verbal IQ, so the parental reports may suffer less from such a bias. In fact, the commonly adopted binary categorization of delay as used in the present study may have actually helped increase sensitivity in discriminating those being atypical in terms of normal milestones from those following a more typical trajectory.

Second, our available structural language measures did not include articulation, intonation, morphology, syntax, complex semantic processing, or other higher order receptive and expressive language abilities. A more comprehensive battery will be needed to fully examine the commonality underlying a broader range of language skills, as well as specific components. In addition, since tasks originally used for estimating aspect(s) of intelligence (VIQ tests) were used here for estimating language abilities, and some language task performance correlated with measured PIQ, the latent “language” factor revealed by PLS might to some extent also reflect a latent factor of general or specific processes of intelligence. The close relationship between language abilities and aspects of measured intelligence (Urbina 2011) should be considered in interpreting the findings.

Third, volumetric approach is not suitable for delineating contributions from geometric features such as surface area and cortical thickness (Ecker, Ginestet et al. 2013), so potential association to these aspects awaits future investigation using surface-based morphometry.

Fourth, this study focuses on individual differences within the autism spectrum rather than how the brain–behavior correlation patterns are different among diagnostic categories, such as neurotypical individuals or individuals with other neurodevelopmental conditions (e.g., specific language impairment, ADHD, intellectual disability). These would be valuable future directions to elucidate the shared and distinct brain–behavior relationships in typical and atypical neurodevelopmental trajectories. An example of this is the noted discrepancy in the association between vocabulary (word knowledge) and local gyrification index within left inferior parietal cortex of male adolescents and young adults with ASC versus matched neurotypical controls (Wallace et al. 2013).

Fifth, due to the substantial heterogeneity within ASC in relation to demographic characteristics (e.g., age and sex) (Duerden et al. 2012; Lai, Lombardo, Suckling et al. 2013) and comorbidities (e.g., intellectual disability, epilepsy, ADHD), whether the findings generalize to other subgroups (e.g., females, children and adolescents, those with intellectual disability or specific language impairments, etc.) must await further investigation.

Lastly, based on the idea that early language development may be a general outcome predictor in human development (Rescorla 2011), one aims of this study is to explore whether in ASC early language development plays a long-lasting role into adulthood in shaping the brain, thus contributing to heterogeneity. Although we have identified brain structures associated with retrospective report of language development, inferences are limited by the correlational nature of the cross-sectional study design, and by associating current neuroanatomy with historical information in a retrospective manner. Adult neuroanatomy may be limited in its ability to inform antecedent causes of the heterogeneity in ASC. Longitudinal studies are needed to prospectively examine if early language development predicts later outcome and heterogeneity in ASC, from neurobiology, behavior to social functioning.

We conclude that both early language development and current language function in adult males with ASC and with average/above-average IQ are associated with variations in current neuroanatomy. Although DSM-5 has put all individuals with autism into a single, umbrella ASD category, it is also important to have explicit recognition of the heterogeneity arising from both developmental and current language (the use of current language specifier for ASD in DSM-5 being an example of this). Both commonality and heterogeneity are key to a better understanding of autism. The clinical and prognostic significance of language-related neuroanatomical variability should be further explored.

## Supplementary Material

Supplementary material can be found at: http://www.cercor.oxfordjournals.org/.

## Funding

This work was supported by the Waterloo Foundation (grant number 921/1247 to S.B.-C. and M.-C.L.), the UK Medical Research Council (grant number GO 400061 to D.G.M.M., S.B.-C., and E.T.B.), and the European Autism Interventions—a Multicentre Study for Developing New Medications (EU-AIMS); EU-AIMS receives support from the Innovative Medicines Initiative Joint Undertaking under grant agreement no. 115300, resources of which are composed of financial contribution from the European Union's Seventh Framework Programme (FP7/2007–2013), from the EFPIA companies in kind contribution and from Autism Speaks. During the period of this work M.-C.L. was supported by the Waterloo Foundation, the Ministry of Education, Taiwan, Wolfson College, Cambridge, EU-AIMS, and the William Binks Autism Neuroscience Fellowship; M.V.L. by the Shirley Foundation, the Wellcome Trust, the British Academy, and Jesus College, Cambridge; B.C. by the UK Medical Research Council; S.B.-C. by the Wellcome Trust, the UK Medical Research Council, the Waterloo Foundation, the Autism Research Trust, and EU-AIMS. This research was also conducted in association with the National Institute for Health Research Collaborations for Leadership in Applied Health Research and Care (NIHR CLAHRC) East of England (EoE). Funding to pay the Open Access publication charges for this article was provided by RCUK and the Wellcome Trust.

## See Appendix for MRC AIMS Consortium Members

The Medical Research Council Autism Imaging Multicentre Study Consortium (MRC AIMS Consortium) is a UK collaboration between the Institute of Psychiatry (IoP) at King's College, London, the Autism Research Centre, University of Cambridge, and the Autism Research Group, University of Oxford. The Consortium members are in alphabetical order: Anthony J. Bailey (Oxford), Simon Baron-Cohen (Cambridge), Patrick F. Bolton (IoP), Edward T. Bullmore (Cambridge), Sarah Carrington (Oxford), Marco Catani (IoP), Bhismadev Chakrabarti (Cambridge), Michael C. Craig (IoP), Eileen M. Daly (IoP), Sean C. L. Deoni (IoP), Christine Ecker (IoP), Francesca Happé (IoP), Julian Henty (Cambridge), Peter Jezzard (Oxford), Patrick Johnston (IoP), Derek K. Jones (IoP), Meng-Chuan Lai (Cambridge), Michael V. Lombardo (Cambridge), Anya Madden (IoP), Diane Mullins (IoP), Clodagh M. Murphy (IoP), Declan G. M. Murphy (IoP), Greg Pasco (Cambridge), Amber N. V. Ruigrok (Cambridge), Susan A. Sadek (Cambridge), Debbie Spain (IoP), Rose Stewart (Oxford), John Suckling (Cambridge), Sally J. Wheelwright (Cambridge), Steven C. Williams (IoP), and C. Ellie Wilson (IoP).

## Supplementary Material

Supplementary Data
