# Supplementary material for: Neuroanatomy of Individual Differences in Language in Adult Males with Autism
Source: Cereb Cortex. 2014 Sep 23;25(10):3613–28. doi: 10.1093/cercor/bhu211 (PMC4585508; doi:10.1093/cercor/bhu211)
Supplement: Supplementary Data [file supp_25_10_3613__index.html]

Neuroanatomy of Individual Differences in Language in Adult Males with Autism — Supplementary Data 

# Neuroanatomy of Individual Differences in Language in Adult Males with Autism

## Supplementary Data

Supplementary Data

**Files in this Data Supplement:**

- Supplementary Data - Doc file
