# Supplementary material for: Neuroanatomy of Individual Differences in Language in Adult Males with Autism
Source: Cereb Cortex. 2014 Sep 23;25(10):3613–28. doi: 10.1093/cercor/bhu211 (PMC4585508; doi:10.1093/cercor/bhu211)
Supplement: Supplementary Data [file supp_bhu211_bhu211supp.doc]

**Supplementary Material**

# Supplementary Text: Previous structural MRI studies on AS vs. HFA

Lotspeich and colleagues found that in boys (aged 7.8-17.9 years), the AS and HFA groups showed distinct patterns in terms of how cerebral gray matter (GM) volume correlated with age and performance IQ. The authors suggested that AS is at a ‘mild’ end of the spectrum in terms of neuroanatomy, and that AS and HFA have neurodevelopmentally distinct brain-behavior relationships . In a small subsample of this cohort (AS n=11, HFA n=9), voxel-based morphometry (VBM) identified decreased GM density in AS compared to HFA in the middle cingulate cortex . In a predominantly male sample aged 7-16 years (AS n=16, HFA n=17), McAlonan and colleagues found that despite having comparable overall total GM volume, increased local GM volume was observed in AS compared to HFA in the basal ganglia, thalamus, posterior cingulate, and precuneus . In a subsequent report (AS n=18, HFA n=18), despite having comparable total white matter (WM) volume, there was decreased local WM volume in AS compared to HFA in the cerebellum and left internal capsule . Finally, in a predominantly male sample of adults (aged 16-59 years) with AS (n=39, full-scale IQ 78-141, mean 106) or ‘autistic disorder’ (n=26, full-scale IQ 53-133, mean 84), Toal and colleagues found decreased local GM volume in AS compared to the autistic disorder group in the right superior temporal lobe and inferior parietal lobule, and increased local WM volume in a small region adjacent to the medial frontal cortex .

Two meta-analyses further provide relevant but indirect information. Using anatomic likelihood estimation, Yu and colleagues contrasted summary findings from VBM studies (ASC vs. controls) with the majority of ASC individuals (>70%) who had a history of language delay with studies of ASC individuals in whom the majority had no language delay. In GM there was a common area in the left ventral posterior temporal cortex where differences were apparent in both sets of studies. However, in all other respects brain areas identified by the two study sets were largely distinct in terms of affected regions and the directionality of differences . The authors could not make a direct comparison between groups with vs. without language delay because there were too few studies providing such data. In another meta-analysis of regional GM volume, Via and colleagues examined the effect of an AS diagnosis vs. other ASC diagnoses. They found no statistically significant effect of ‘diagnosis’ and concluded that AS and autistic disorder share similar neural substrates . However, the authors limited their analysis to brain regions showing significant differences between all individuals with ASC and controls, which may have missed significant differences outside of these confined regions.

# Supplementary Tables

**Supplementary Table S1. VBM group-differences^1^ for “neurotypical (NT) vs. ASC with language delay (ASC+D)” and “NT vs. ASC without language delay (ASC+nD)”**

| **Region** | **Cluster size (voxels)** | **Cluster-wise FDR *q*** | **Peak-voxel T** | **Peak-voxel MNI coordinate** |
| --- | --- | --- | --- | --- |
| ***GM*** |  |  |  |  |
| **NT > ASC+D** |  |  |  |  |
| Cerebellum (right) | 4235 | 0.009 | 5.96 | 57 -55 -36 |
| Anterior temporal lobe (right) | 7960 | <0.001 | 5.29 | 51 12 -39 |
| Thalamus (left) | 6281 | 0.001 | 4.11 | -9 -27 10 |
| Thalamus, putamen, amygdala, hippocampus, insula (right) | 12996 | <0.001 | 3.87 | 15 -18 9 |
| Insula, putamen, amygdala, hippocampus (left) | 4331 | 0.009 | 3.81 | -39 -0 9 |
| Cerebellum (left) | 6567 | 0.001 | 3.55 | -42 -43 -31 |
|  |  |  |  |  |
| **NT > ASC+nD** |  |  |  |  |
| Cerebellum (right) | 5686 | 0.008 | 3.98 | 50 -51 -41 |
|  |  |  |  |  |
| **ASC+D > NT** |  |  |  |  |
| Pons, medulla oblongata | 3581 | 0.031 | 5.69 | -15 -22 -36 |
| Postcentral and supramarginal gyri (left) | 6469 | 0.001 | 5.10 | -44 -29 49 |
| Dorsolateral and dorsomedial prefrontal cortices (left & right) | 20582 | <0.001 | 4.96 | -25 32 47 |
| Inferior parietal lobule (left) | 2977 | 0.043 | 4.53 | -47 -58 39 |
| Precuneus (left & right) | 2980 | 0.043 | 4.14 | -6 -57 52 |
| Posterior temporal cortex (left) | 6566 | 0.001 | 4.10 | -59 -60 3 |
| Temporo-parietal junction (right) | 3321 | 0.036 | 3.90 | 60 -50 27 |
|  |  |  |  |  |
| **ASC+nD > NT** |  |  |  |  |
| Heschl gyrus, superior temporal gyrus (right) | 6726 | 0.002 | 4.45 | 47 -16 2 |
| Temporo-occipital junction (inferior temporal gyrus) (left) | 4312 | 0.015 | 4.04 | -51 -67 -17 |
| Angular gyrus, temporo-parieto-occipital junction (left) | 5648 | 0.004 | 3.92 | -47 -59 38 |
| Heschl gyrus, superior temporal gyrus (left) | 3482 | 0.036 | 3.42 | -42 -33 15 |
|  |  |  |  |  |
| ***WM*** |  |  |  |  |
| **ASC+D > NT** |  |  |  |  |
| Posterior frontal and anterior parietal regions (right) | 10081 | 0.001 | 4.42 | 42 -17 36 |

^1^ Thresholded at voxel-level *p* < 0.025 and cluster-level topological FDR *q* < 0.05.

# Supplementary References

Kwon H, Ow AW, Pedatella KE, Lotspeich LJ, Reiss AL. 2004. Voxel-based morphometry elucidates structural neuroanatomy of high-functioning autism and Asperger syndrome. Dev Med Child Neurol. 46:760-764.

Lotspeich LJ, Kwon H, Schumann CM, Fryer SL, Goodlin-Jones BL, Buonocore MH, Lammers CR, Amaral DG, Reiss AL. 2004. Investigation of neuroanatomical differences between autism and Asperger syndrome. Arch Gen Psychiatry. 61:291-298.

McAlonan GM, Cheung C, Cheung V, Wong N, Suckling J, Chua SE. 2009. Differential effects on white-matter systems in high-functioning autism and Asperger's syndrome. Psychol Med. 39:1885-1893.

McAlonan GM, Suckling J, Wong N, Cheung V, Lienenkaemper N, Cheung C, Chua SE. 2008. Distinct patterns of grey matter abnormality in high-functioning autism and Asperger's syndrome. J Child Psychol Psychiatry. 49:1287-1295.

Toal F, Daly EM, Page L, Deeley Q, Hallahan B, Bloemen O, Cutter WJ, Brammer MJ, Curran S, Robertson D, Murphy C, Murphy KC, Murphy DG. 2010. Clinical and anatomical heterogeneity in autistic spectrum disorder: a structural MRI study. Psychol Med. 40:1171-1181.

Via E, Radua J, Cardoner N, Happe F, Mataix-Cols D. 2011. Meta-analysis of gray matter abnormalities in autism spectrum disorder: should Asperger disorder be subsumed under a broader umbrella of autistic spectrum disorder? Arch Gen Psychiatry. 68:409-418.

Yu KK, Cheung C, Chua SE, McAlonan GM. 2011. Can Asperger syndrome be distinguished from autism? An anatomic likelihood meta-analysis of MRI studies. J Psychiatry Neurosci. 36:100138.
